# Biomarkers of systemic inflammation in farmers with musculoskeletal disorders; a plasma proteomic study

**DOI:** 10.1186/s12891-016-1059-y

**Published:** 2016-05-10

**Authors:** Bijar Ghafouri, Anders Carlsson, Sara Holmberg, Anders Thelin, Christer Tagesson

**Affiliations:** Division of Community Medicine, Department of Medical and Health Sciences, Faculty of Health Sciences, Linköping University and Pain and Rehabilitation Center, Anaesthetics, Operations and Specialty Surgery Center, Region Östergötland, Linköping, SE-581 85 Sweden; Division of Neuro and Inflammation Science, Department of Clinical and Experimental Medicine, Linköping University, Occupational and Environmental Medicine Center, Heart and Medicine Center, Region Östergötland, Linköping, Sweden; Department of Research and Development, Region Kronoberg, Växjö, Sweden; Department of Public Health and Caring Sciences, Family Medicine and Clinical Epidemiology Sections, Uppsala University, Uppsala, Sweden

**Keywords:** Musculoskeletal disorder, Farmers, Proteomic, Systemic inflammation, Occupational medicine

## Abstract

**Background:**

Farmers have an increased risk for musculoskeletal disorders (MSD) such as osteoarthritis of the hip, low back pain, and neck and upper limb complaints. The underlying mechanisms are not fully understood. Work-related exposures and inflammatory responses might be involved. Our objective was to identify plasma proteins that differentiated farmers with MSD from rural referents.

**Methods:**

Plasma samples from 13 farmers with MSD and rural referents were included in the investigation. Gel based proteomics was used for protein analysis and proteins that differed significantly between the groups were identified by mass spectrometry.

**Results:**

In total, 15 proteins differed significantly between the groups. The levels of leucine-rich alpha-2-glycoprotein, haptoglobin, complement factor B, serotransferrin, one isoform of kininogen, one isoform of alpha-1-antitrypsin, and two isoforms of hemopexin were higher in farmers with MSD than in referents. On the other hand, the levels of alpha-2-HS-glycoprotein, alpha-1B-glycoprotein, vitamin D- binding protein, apolipoprotein A1, antithrombin, one isoform of kininogen, and one isoform of alpha-1-antitrypsin were lower in farmers than in referents. Many of the identified proteins are known to be involved in inflammation.

**Conclusions:**

Farmers with MSD had altered plasma levels of protein biomarkers compared to the referents, indicating that farmers with MSD may be subject to a more systemic inflammation. It is possible that the identified differences of proteins may give clues to the biochemical changes occurring during the development and progression of MSD in farmers, and that one or several of these protein biomarkers might eventually be used to identify and prevent work-related MSD.

**Electronic supplementary material:**

The online version of this article (doi:10.1186/s12891-016-1059-y) contains supplementary material, which is available to authorized users.

## Background

Farmers in industrialized countries have low morbidity in general and common disease entities as cancer and cardiovascular disorders are less frequent among farmers than in the general population [1]. By contrast, farmers have an increased risk of acquiring musculoskeletal disorders (MSD), such as osteoarthritis of the hip, low back pain, and neck and upper limb complaints [1–3]. The reason for this is not known. We have previously demonstrated that the increased risk cannot be fully explained by known risk factors such as physical work load, psychosocial factors, or life style, indicating that other etiological factors must be involved. [2–4]. Interestingly, the prevalence of low back pain in farmers is associated not only with other musculoskeletal symptoms but also with chest discomfort, dyspepsia, symptoms from eyes, nose and throat mucous membranes, skin problems, work-related fever attacks, and primary care appointments due to digestive disorders [4]. This comorbidity points to the possibility that MSD in farmers may be part of a more systemic disorder with pathological changes in many organs and tissues. Since blood is in direct contact with all tissues, such changes are likely to be reflected in plasma [5] and quantitative changes in the plasma could thus serve as biomarkers of systemic effects. Moreover, plasma samples are readily available for investigation, thus making analysis of plasma a favorable alternative for examining laboratory signs of a systemic disorder. In a recent study serum samples from patient with discogenic chronic low back pain (DLBP) were analyzed by mass spectrometry to identify biomarkers for DLBP [6].

In this study, our objective was to identify plasma proteins that might differentiate farmers with MSD from matched rural referents. We focused on relatively abundant plasma proteins which can be quantified to the nanogram range using two-dimensional gel electrophoresis (2DE) with silver staining and subsequent identification by mass spectrometry (MS).

## Methods

### Subjects

The investigated subjects were selected from a prospective study cohort established with the intention to study health promoting factors related to farming and lifestyle [7]. All occupationally active male farmers born between 1930 and 1949 in nine rural municipalities in Sweden were identified in the Swedish National Farm Register and included in the cohort. The cohort represents a variety of farm types and geographical variation. The areas were chosen with consideration to known east-west and north-south cardiovascular disease gradients in the Swedish population [8] and to represent a variety of farm types and geographical variation over the country. Farmers were defined as men who owned or rented a farm and who spent at least 25 h per week farming. The occupational activity was checked with local representatives from the Federation of Swedish Farmers. To each farmer a rural referent, matched by age, sex and residential area, was sampled from the National Population Register. The rural referents were to be occupationally active in other than farming. Altogether 1220 farmers and 1130 rural non-farmers were eligible and included in the cohort. The farmers and the rural referents (*n* = 2350) were invited to an extensive health survey including questionnaires, interviews, physical examinations and laboratory test [9].

The cross-sectional population-based survey identified farmers with MSD using a questionnaire with a number of items addressing symptoms from back, hip, knee, neck, forearms and shoulders. Information on symptoms from the musculoskeletal system was assessed by self-administrated questionnaire [3]. The subjects were asked to indicate (yes or no) if they had ever (more than occasionally) had pain, aches or discomfort in the neck-shoulder area, the low back, hips or knees. The questionnaire file shows this in more detail (see Additional file 1). Similar symptoms during the last year were also indicated. The subjects were asked to indicate whether they had sought medical advice or treatment for the various symptoms, and concerning low back and knee symptoms, whether they had been on sick leave due to the illness. A large number of farmers and non-farmers (89 %) reported symptoms from musculoskeletal system. The farmers reported significantly more symptoms from low back and hips [4, 9].

This large material was used to identify farmers with symptoms from the musculoskeletal system only. Thirteen farmers working with swine confinement and cattle raising and their rural referents (Table 1) were selected and included in this explorative hypothesis generated study.

**Table 1:** Musculoskeletal symptoms among farmers and referents included in this study

Musculoskeletal symptoms	Farmers	Referents	*p*-value
	*N* = 13	*N* = 11	
Neck and shoulder pain	9	6	ns
Hands and forearms(numbness)	4	4	ns
Hands and forearms(nocturnal pain)	1	2	ns
Low back pain	6	3	ns
Ischialgia	5	1	ns
Hips problems	10	5	ns
Groin-thigh pain	4	3	ns
Knee joint problems	8	2	ns
All symptoms	13	5	0.007

### Sample preparation

All samples were blinded before analysis. Plasma from farmers and referents were prepared at the same time and not separately. 50 μl of plasma was applied onto an albumin & IgG Depletion column (GE Healthcare). Albumin and IgG were removed according to the manufacturer’s recommendation. The samples were then desalted and lyophilized. The protein concentration was determined according to Bradford [10].

### 2-DE analysis

The lyophilized proteins were dissolved in 0.20 ml urea sample solution according to Görg [11]. 2-DE was performed in a horizontal 2-DE setup (IPGphore and Multiphore from GE Healthcare), as described in detail previously [12] and essentially according to Görg [11]. The samples (containing 50 μg protein for analytical gels and 900 μg for preparative gels) were applied by in-gel rehydration (according to the manufacturer’s instructions) for 12 h using low voltage (30 V) in pH 4–7 IPGs. The proteins were then focused for up to 32 000 Vhs at a maximum voltage of 8000 V. IPGs were either used immediately for second dimensional analysis, or stored at −70 °C until analyzed. The second dimension (SDS-PAGE) was carried out by transferring the proteins to gradient gels cast on GelBond PAG film (0.5/180/245 mm, 11–18%T, 1.5%C, 33–0 % glycerol) running at 30 mA and up to 1000 V for about 5 h. In analytical gels, separated proteins were detected by silver staining according to Schevchenko as described previously [12]. The preparative gels, for MS analysis, were fluorescently stained with SYPRO Ruby [12]. All staining and washing steps were performed with continuous gentle agitation. The protein patterns were analyzed as digitized images, using a CCD camera in combination with a computerized imaging 12-bit system designed for evaluation of 2-DE patterns. The amount of protein in a spot was assessed as background corrected optical density, integrated over all pixels in the spot and expressed as integrated optical density (IOD). In order to correct for differences in total silver stain intensity between different 2-DE images, the amounts of the compared protein spots were quantified as optical density for individual spot per total protein intensity of all spots in the same gel. Thereby ppm-values (parts per million) for all proteins were generated that were evaluated for differences between the groups.

### Protein identification

Protein spots were excised using a homemade spot picker. The picked protein spots were digested with trypsin (Promega/SDS Biosciences, Falkenberg, Sweden) as described previously [13]. The trypsinated peptides were applied to mass spectrometry for protein identification. The dried tryptic samples from fluorescently stained proteins were dissolved in 6 μl of 0.1 % formic acid. Peptides were analyzed using an on-line nano-flow HPLC system (EASY-nLC; Proxeon, Bruker Daltonics) in conjugation with the mass spectrometer HCTultra PTM Discovery System (Bruker Daltonics) as previously described [13]. LC-MS/MS spectra were processed by Bruker Daltonics DataAnalysis 3.4 (Bruker Daltonics, Bremen, Germany) and resulting MS/MS data were searched in NCBInr and Swiss-Prot database on MASCOT server: www.matrixscience.com Database search parameters were set as follows: the enzyme trypsin was used; up to one missed cleavage was allowed; fixed modification included were carbamidomethylation of cysteine and oxidation of methionine; mass tolerance for MS precursor ion was 0.8 Da and for MS/MS fragment ion was 0.6 Da; and charge states were varied. Criteria for identification of a protein were at least 3 peptides of the protein should be identified with a MASCOT score over 25 and an expectation value <1. The mass list generated from the major peaks of the MALDI spectra was submitted to a database search (NCBI or SWISS-PROT) using MS-FIT search engines. Restrictions were placed on species (Human), mass tolerance (50 ppm), maximum missed cleavages by trypsin (up to 1) and cysteine modification by carbamidomethylation.

### Statistical analysis

Non-parametric Mann-Whitney *U* test was used as statistical method to calculate significant differences between groups. A *p* < 0.05 was considered statistically significant. All results are given as mean ± standard deviation. IBM SPSS Statistics 20 was used as statistical software.

## Results

### 2-DE analysis and mass spectrometry

**Fig. 1:**
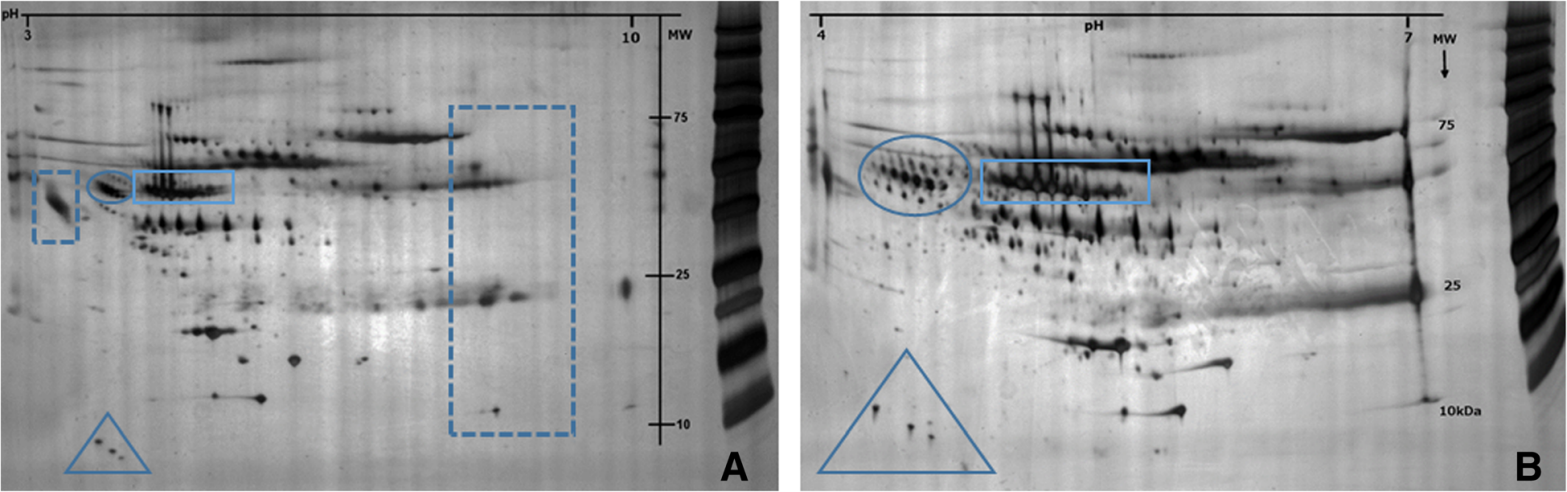
Comparison between plasma protein separation in pI range of 3–10 (**a**) and 4–7 (**b**). The marked areas are some example of the improved resolution using IPGs 4–7

**Fig. 2:**
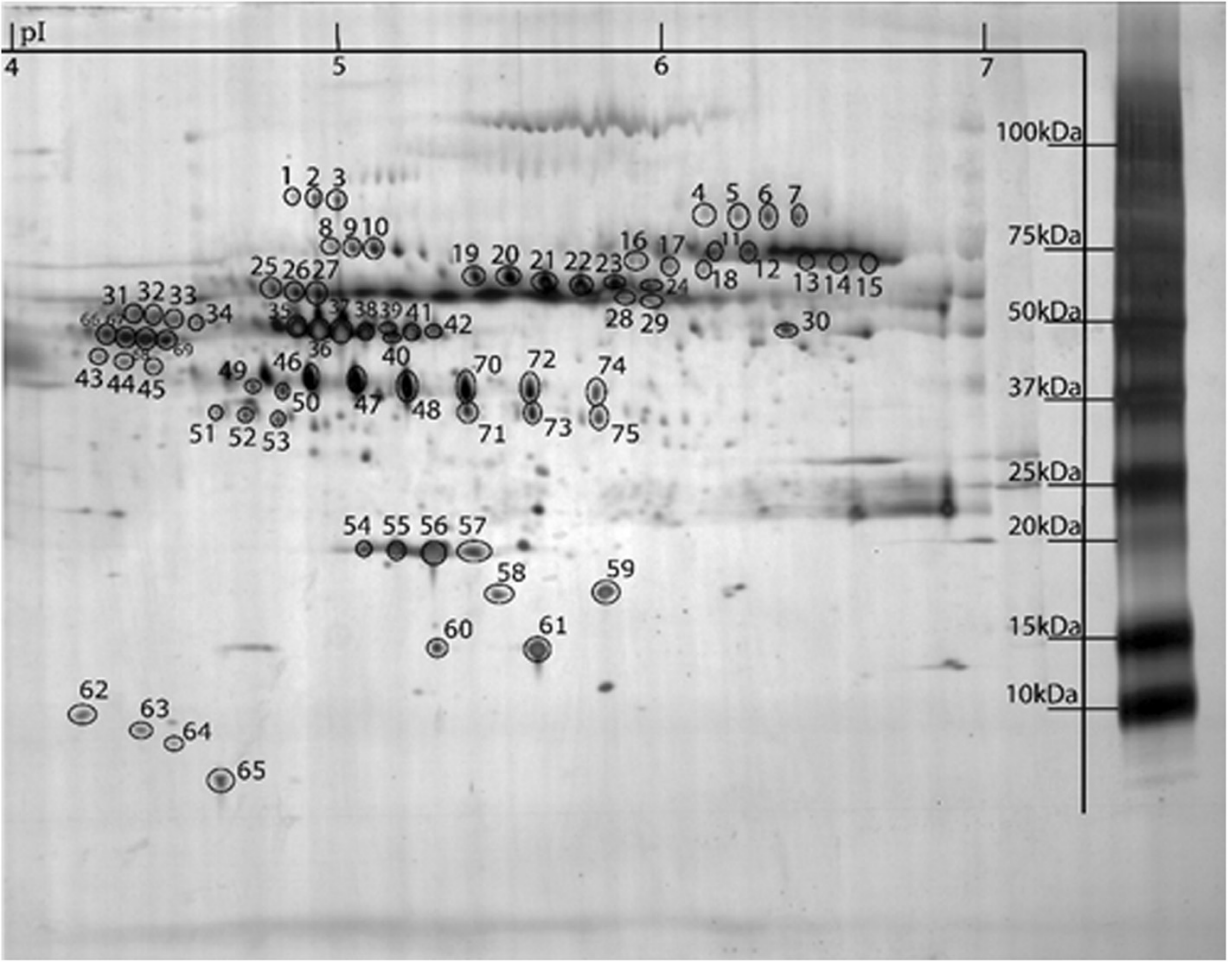
A typical 2DE protein pattern of human plasma in pI range of 4–7. The marked spots refer to identified proteins in Table 1

To determine proper 2DE conditions for the analysis to be made, plasma proteins were separated in the pI ranges 3–10 and 4–7 (first dimension) followed by SDS-PAGE (second dimension). As illustrated in Fig. 1, most of the spots detected in pI range 3–10 were also detected in the pI range 4–7 (about 90 %). The marked areas in Fig. 1 illustrate the good resolving power when using IPGs in pI range 4–7 compared to 3–10. A few proteins (Fig. 1a, dashed squares9 with pI outside the 4–7 range were lost. Due to the good separation in the pI range 4–7 we decided to analyze all samples using IPGs 4–7. Mapping of the plasma proteome in the pI range 4–7 was performed by mass spectrometry. Seventy five spots could be identified (Table 2, Fig. 2). According to the accession number these identified protein spots represent 20 different proteins. Several of the identified proteins were expressed as different isoforms due to post- translational modifications such as glycosylation and phosphorylation.

**Table 2:** Identified proteins in plasma by nLC-MS/MS

Spot Nr	Protein	SwissProt	Mw/pI	Score	Sequence coverage [%]	Nr of unique peptides
1	Alpha-1-antitrypsin	P01009	46878 / 5.37	194	21	7
2	Alpha-1-antitrypsin			474	33	12
3	Alpha-1-antitrypsin			382	32	15
4	Complement factor B	P00751	86847 / 6.67	225	12	10
5	Complement factor B			266	16	12
6	Complement factor B			339	15	15
7	Complement factor B			252	15	14
8	Alpha-1B-Glycoprotein	P04217	54790 / 5.56	51	1	6
9	Alpha-1B-Glycoprotein			108	7	3
10	Alpha-1B-Glycoprotein			142	12	5
11	Serotransferrin	P02787	79294 / 6.81	414	19	13
12	Serotransferrin			988	52	31
13	Serotransferrin			889	42	28
14	Serotransferrin			1107	49	31
15	Serotransferrin			1078	49	31
16	N-acetylmuramoyl-L-alanine amidase	Q96PD5	62748 / 7.25	31	1	1
17	N-acetylmuramoyl-L-alanine amidase			67	3	2
18	N-acetylmuramoyl-L-alanine amidase			98	3	1
19	Hemopexin	P02790	52385 / 6.55	212	14	6
20	Hemopexin			306	23	11
21	Hemopexin			314	26	12
22	Hemopexin			329	25	11
23	Hemopexin			298	15	10
24	Hemopexin			277	20	9
25	Kininogen	P01042	72996 / 6.34	321	11	6
26	Kininogen			308	9	5
27	Kininogen			203	10	6
28	Hemopexin	P02790	52385 / 6.55	266	20	8
29	Hemopexin			49	2	1
30	Beta-2-Glycoprotein 1	P02749	39584 / 8.34	172	16	5
31	Kininogen	P01042	72996 / 6.34	127	8	4
32	Kininogen			275	17	8
33	Kininogen			236	13	8
34	Kininogen			254	15	8
35	Alpha-1-antitrypsin	P01009	46878 / 5.37	962	45	17
36	Alpha-1-antitrypsin			730	43	18
37	Alpha-1-antitrypsin			1009	47	17
38	Alpha-1-antitrypsin			509	42	16
39	Vitamin D-binding protein	P02774	54526 / 5.40	476	36	16
40	Alpha-1-antitrypsin	P01009	46878 / 5.37	620	51	19
41	Vitamin D-binding protein	P02774	54526 / 5.40	381	25	11
42	Antithrombin III	P01008	53025 / 6.32	333	25	10
43	Leucine-rich alpha-2-glycoprotein	P02750	38382 / 6.45	224	17	6
44	Leucine-rich alpha-2-glycoprotein			131	14	5
45	Leucine-rich alpha-2-glycoprotein			134	17	4
46	Haptoglobin	P00738	45861 / 6.13	302	21	9
47	Haptoglobin			273	26	10
48	Haptoglobin			296	27	10
49	Zinc-Alpha-2-glycoprotein	P25311	34465 / 5.71	223	17	7
50	Zinc-Alpha-2-glycoprotein			226	24	8
51	Clusterin	P10909	53031 / 5.89	219	17	7
52	Clusterin			203	14	7
53	Clusterin			331	22	9
54	ApoA1	P02647	30759 / 5.56	419	44	13
55	ApoA1			775	62	16
56	ApoA1			4287	53	14
57	ApoA1			850	48	8
58	Haptoglobin	P00738	45861/6.13			2
59	Haptoglobin					2
60	Transthyretin	P02766	15887/ 5.5	4357	69.4	11
61	Transthyretin			8483	64.6	7
62	ApoCIII	P02656	8800/4.7		65	4
63	ApoCIII				65	4
64	ApoAII	P02652	9300/6.5		56	5
65	ApoCII	P02655	8900/4.7		96	6
66	Alpha-2-HS-Glycoprotein	P02765	39325/5,4	60245	22.9	9
67	Alpha-2-HS-Glycoprotein			60245	20.4	8
68	Alpha-2-HS-Glycoprotein			60245	20.4	8
69	Alpha-2-HS-Glycoprotein			60245	20.4	8
70	Haptoglobin	P00738	45861/6.13	14986	24.1	11
71	Haptoglobin			12798	23.2	8
72	Haptoglobin			12728	21.9	8
73	Haptoglobin			60393	28.3	11
74	Haptoglobin			12798	23.2	8
75	Haptoglobin			12728	21.9	8

The spot numbers refer to marked protein spots in Fig. 1

### Protein changes in farmers with MSD compared to rural referents

**Fig. 3:**
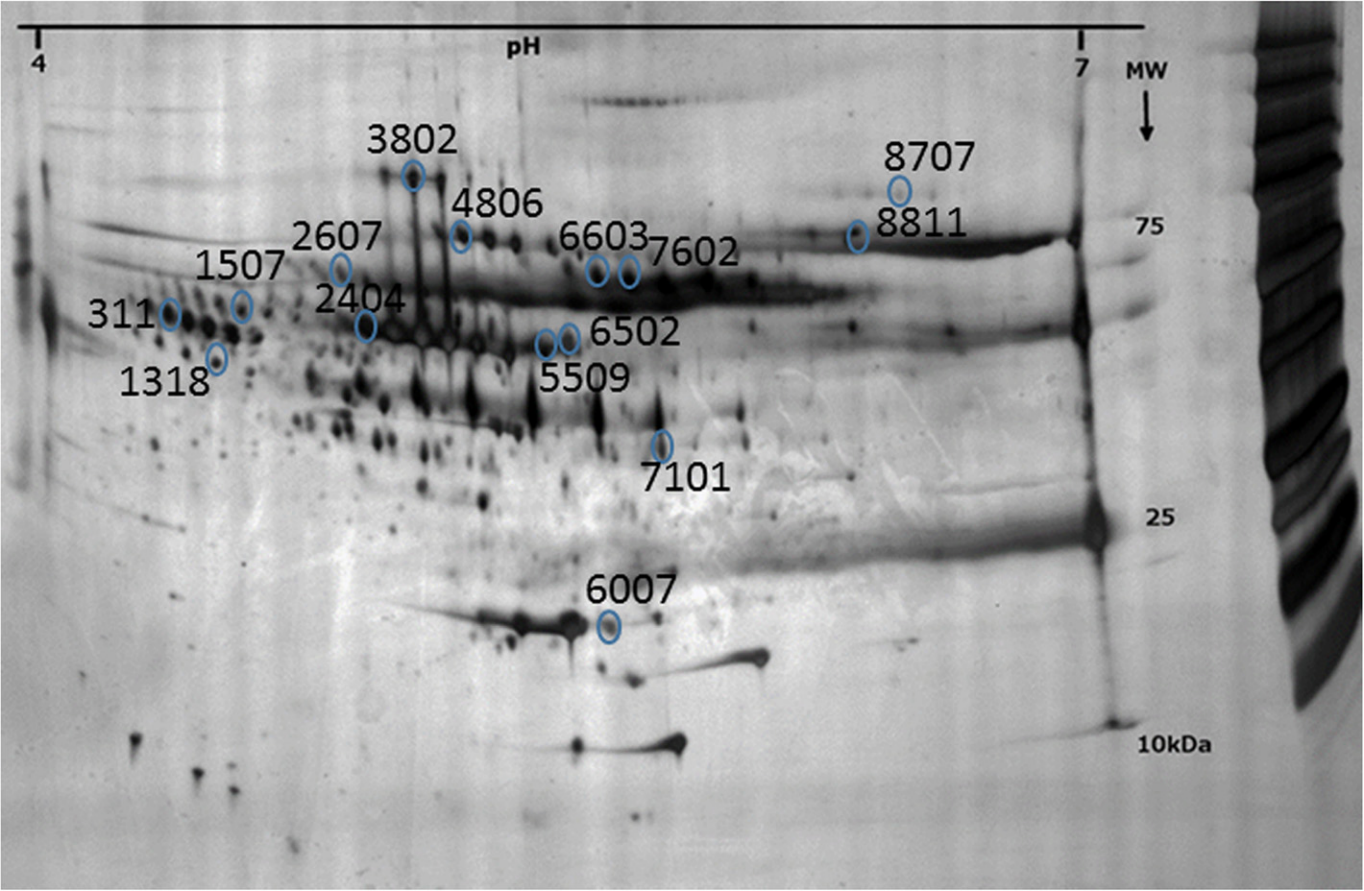
Significantly changed proteins in farmers with musculoskeletal disorders compared to rural referents

**Fig. 4:**
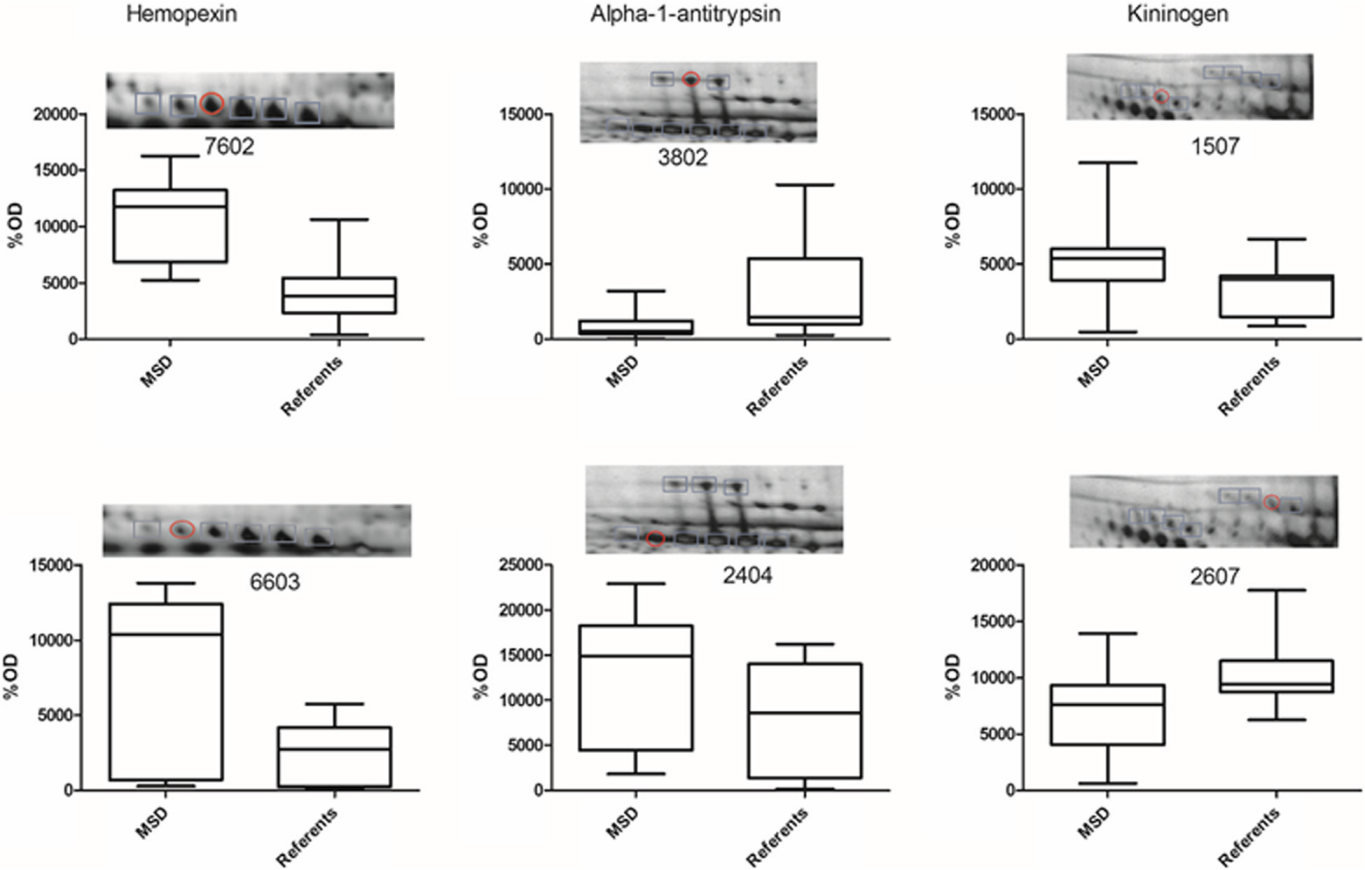
Quantitative data for protein isoforms that significantly (*p* < 0.05) changed in farmers with musculoskeletal disorders compared to rural referents. The blue marked spots are the different isoforms and the red circle is the significantly changed protein

More than 200 protein spots were detected in all samples. To investigate possible differences in the plasma proteomes of farmers with MSD and rural referents, protein spots present in at least 50 % of the gels in either group were matched, quantified for intensity, and compared between the groups. Proteins whose concentrations were statistically different in the two groups were then identified using mass spectrometry. As shown in Table 3, the levels of leucine-rich alpha-2-glycoprotein, haptoglobin, complement factor B, serotransferrin, one isoform of kininogen (spot1507), one isoform of alpha-1-antitrypsin (spot 2404), and two isoforms of hemopexin (spots 6603 and 7602) were higher in farmers with MSD than in rural referents. On the other hand, the levels of alpha-2-HS-glycoprotein, alpha-1B-glycoprotein, vitamin D- binding protein, apolipoprotein A1, antithrombin, one isoform of kininogen (spot 2607), and one isoform of alpha-1-antitrypsin (spot 3802) were lower (Table 2). Figure 3 pinpoints the statistically differing proteins on a 2DE gel, while Fig. 4 illustrates how the different isoforms of kininogen, alpha-1-antitrypsin, and hemopexin were quantitatively expressed in farmers with MSD and referents, respectively.

**Table 3:** Proteins that were present differently in the plasma of farmers with MSD and their matched rural referents (RR)

Spot	Protein	MSD *vs* RR	Accession number	OD (MSD) Mean ± (SD) × 10^3^	OD (RR) Mean ± (SD) × 10^3^	*p*-value
311	Alpha-2-HS-Glycoprotein	↓	P02765	4.768 (3.556)	7.720 (2.386)	0.046
1318	Leucine-rich alpha glycoprotein	↑	P02750	4.587 (3.355)	2.081 (1.285)	0.04
1507	Kininogen	↑	P01042	5.288 (2.631)	3.425 (1.684)	0.049
2404	Alpha1-anti trypsin	↑	P01009	12.938 (7.270)	7.799 (6.398)	0.04
2607	Kininogen	↓	P01042	6.885 (3.786)	10.114 (2.964)	0.03
3802	Alpha1-anti trypsin	↓	P01009	0.884 (0.840)	3.117 (3.168)	0.019
4806	Alpha-1B-glycoprotein	↓	P04217	4.677 (2.359)	6.487 (1.138)	0.01
5507	Vitamin D binding protein	↓	P02774	5.506 (2.400)	8.585 (2.693)	0.03
6007	Apolipoprotein A1	↓	P02647	3.765 (3.513)	6.838 (2.857)	0.010
6502	Antithrombin III	↓	P01008	1.940 (1.750)	5.128 (2.950)	0.005
6603	Hemopexin	↑	P02790	7.613 (5.647)	2.377 (2.225)	0.019
7101	Haptoglobin	↑	P00738	5.455 (1.552)	3.573 (1.382)	0.008
7602	Hemopexin	↑	P02790	10.729 (3.657)	4.121 (2.744)	0.000
8707	Complement factor B	↑	P02790	2.468 (2.920)	1.226 (2.029)	0.022
8811	Serotransferrin	↑	P02787	8.633 (3.460)	5.355 (2.047)	0.010

The spot numbers refer to the marked spots in Fig. 3

Arrow up (↑) indicates up-regulated and arrow down (↓) down-regulated proteins in farmers with MSD compared to RR

## Discussion

Using 2-DE and mass spectrometry proteomic techniques, we were able to identify 15 proteins that were present differently in the plasma of farmers with MSD and their matched rural referents. Several of the proteins with plasma levels higher among farmers than among referents are well known as mediators or indicators of inflammation. For example*, complement factor B* is essential for complement activation via the alternate pathway and so plays an important role in inflammatory conditions such as arthritis [14–16] and muscle inflammation [17]. *Haptoglobin* is established as an inflammation-inducible plasma protein [18] that exerts a regulatory activity on immune cell responses and host immunity [19]. Accordingly, haptoglobin was found to be a monocyte chemoattractant whose chemotactic potential was mediated, at least in part, by its interaction with chemokine (C-C motif) receptor 2 [20]. Recently, Park and co-workers demonstrated that the C-terminal haptoglobin fragment is generated by plasmin in local inflammatory environments, suggesting that this fragment might be applied as a novel biomarker for the diagnosis and prognosis of inflammatory joint diseases such as rheumatoid arthritis [21]. *Hemopexin* belongs to the acute phase reactants, the synthesis of which is induced after inflammation. Aside from a systemic role in preventing heme-mediated oxidative stress, hemopexin might act as a local antioxidant at sites of injury and so play a role in inflammation [22].

Analogously, some of the proteins with plasma levels lower among the farmers than among the referents (Table 3) are known to have anti-inflammatory properties and actions. Thus, *antitrombin III* has been shown to inhibit cytokine and tissue factor production in endothelial cells [23], attenuate CD11b/CD18 expression in activated neutrophils and monocytes [24], suppress granule secretion in activated platelets [25], prevent apoptosis of hepatocytes [26, 27], and improve endotoxin-induced acute lung injury in rats [28]. *Alpha-2-HS-glycoprotein* a multifunctional molecule that may serve as an inhibitor of systemic inflammation in mice [29] and humans [30, 31]; notably, its serum levels were significantly decreased in patients with RA [32, 33]. V*itamin D-binding protein (VDBP)* is primarily known as a transport protein but was found to be down-regulated in serum of experimental animals with systemic inflammation [34] and patients with sepsis [35, 36]. Moreover, Oh and coworkers recently found this protein to be down-regulated in serum of patients with carpal tunnel syndrome [37], a condition causing pain, impairment, and disability. The molecular mechanisms connecting inflammation with decreased VDBP are not clear, but because VDBP is a precursor for macrophage activating factor (MAF) it may be speculated that the decreased levels reflect increased formation of MAF and ensuing activation of macrophages [38] and neutrophils [39] at sites of inflammation. Alternatively, MAF may function positively by causing death of the macrophages when activated macrophages are no longer needed at the site of inflammation [40] Clearly, much further investigation is needed to elucidate why farmers with MSD appear to have decreased serum levels of VDBP.

One of the most important aspects of using 2DE is the capability of resolving many post-translationally modified proteins that may appear as isoforms. We identified two isoforms of hemopexin that both were present in higher amounts in the plasma of farmers (Table 3, Fig. 4). Moreover, we identified two proteins (kininogen and alpha-1-antitrypsin) that were present differently in the plasma of farmers and referents depending on isoform, i.e., one isoform was present in a higher amount in the farmer group *vs* the referent group, and another isoform was present in a higher amount in the referent group *vs* the farmer group (Table 3, Fig. 4). While we are unable to conclusively explain these findings, it can be noted that these proteins, too, are being associated with inflammatory processes and diseases. Thus, *kininogen* is the precursor of bradykinin, a well-known mediator of inflammation and pain [41–43] that plays a crucial role in rheumatoid arthritis [44]. *Alpha-1-antitrypsin* can exert anti-inflammatory activity in many tissues and organs including the musculoskeletal system; notably, low levels of alpha-1-antitrypsin have been suggested to contribute to the development of rheumatoid arthritis [45] and MSD [46]. Yet, changes in isoforms of kininogen, alpha-1-antitrypsin, and hemopexin have not previously been reported. We do not know the exact chemical nature of the isoforms found in our study and are currently attempting to ascertain post-translational modifications. Characterization of the chemical nature of these protein isoforms, determination of the tissue(s) that produce them, and the relationship between these isoforms and other interacting proteins—all of these, as they relate to the causal events of MSD, may lead to a further understanding of the progression to MSD in farmers.

While the levels of leucine-rich alpha-2- glycoprotein were higher in the farmer group, the levels of alpha-1B-glycoprotein were higher in the referent group (Table 3). Whether these proteins, too, are involved in inflammation or not remains unclear. The physiological role of *leucine-rich alpha-2- glycoprotein* is not known; however, its 33 % sequence homology with a beta-type phospholipase A2 inhibitor [47] suggests the possibility that it might modulate inflammation and pain through inhibition of phospholipase A2 activity. As to *alpha-1B-glycoprotein*, decreased plasma levels of this protein were found in patients with rheumatoid arthritis [32] but little is known about the function of this protein and the biological relevance of this finding is therefore uncertain. The reason why *serotransferrin* (the major iron-transporting plasma protein) was higher in the farmer group is also uncertain; interestingly, its plasma levels were affected in an experimental model of neuropathic pain [48]. *Apolipoprotein A1* (ApoA1) has been shown to have anti-inflammatory properties and to reduce atherosclerosis [49]; one would expect, therefore, that farmers (who are at lower risk for cardiovascular disease than the general population) had higher (rather than lower) apoA1 than referents. On the other hand, the level of apoA1 in lipoproteins has been found to correlate positively with PCYOX1 [50], an enzyme which generates H_2_O_2_ and so probably plays a role in atherogenesis [51]*.* Based on this finding, Sun and coworkers suggested that high apoA1 content might be associated with increased risk of cardiovascular disease [50]. Further investigation is required to clarify the reason why farmers with MSD appear to have decreased serum levels of apo A1.

Although we were able to identify a protein profile that differentiates farmers with MSD from referents using proteomic techniques, the methods used in this study may pose some limitations. We used isoelectric points and molecular weight of the proteins to separate the proteins onto a two-dimensional gel. We were limited therefore to investigating proteins within a certain range of isoelectric points and molecular weight, thereby excluding several proteins that may be potential candidates for biomarkers of MSD in farmers. Furthermore, we used a depletion column to remove the high abundant proteins (albumin and IgG) before analysis these proteins give substantial background to the 2DE profiles and make analysis difficult [5]; it is not impossible that this procedure might have eliminated some other candidates. As to the protein resolution, we have little reason to believe that the methods used have posed further limitations; rather, an advantage of 2DE compared to other separation methods is the quality of protein resolution [52] as shown by the capability of resolving many post-translationally modified proteins that may appear as isoforms. Identity of each protein spot was determined by mass spectrometry, which is the method of choice for accurate probability based protein identification. Moreover, it is unlikely that the results are flawed by unacceptable analytical errors; densitometric analyses combined with silver staining of proteins are highly quantitative and a median coefficient of variation of approximately 8 % has been reported using similar parameters as used in our procedure (i.e., fluorescent staining, PDQuest software) [53].

## Conclusions

In conclusion, we have identified a number of proteins that are present differently in the plasma of farmers with MSD and their rural referents. Many of these proteins are known to be mediators or indicators of inflammation, supporting the hypothesis that farmers with MSD may be subject to a more systemic inflammation. The implication of these findings is worth considering. First, it is possible that the identified proteins may give clues to the biochemical changes occurring during the development and progression of MSD in farmers and so contribute to a deeper understanding of the pathogenetic mechanisms behind MSD and related symptoms/findings in other organ systems. Second, one or several or a combination of the identified proteins might possibly be used as a biomarker(s) of MSD in farmers. Much further investigation is therefore warranted, including comparisons of farmers with and without MSD and referents with and without MSD to elucidate how MSD protein picture relates to exposure. Third, if it turns out that a plasma biomarker(s) appears early during the development of MSD in farmers, this biomarker(s) might eventually be used to identify farmers who are at greater risk and to prevent progression of the disease process.

This study provides novel information about the biological mechanisms behind MSD in farmers and makes it possible to explain the link between health outcomes and the work environment. We identify biomarkers of systemic inflammation that contribute to better understanding of the pathophysiological processes involved in MSD. Although farmers are a relatively small occupational group in Sweden today, the identification of the causes of work-related MSD in this group may be a valuable tool for understanding of similar phenomenon in other groups. The results of this study thus may contribute to a better understanding of how problems in the musculoskeletal system generally arise.

## Ethics approval and consent to participate

The cohort study has been approved by the Research Ethics Committee at the Karolinska Institute in Stockholm, Sweden, and by the Regional Ethics Board in Uppsala, Sweden. All men who participated in the health surveys gave their informed consent.

## Consent for publication

Not applicable.

## Availability of data and materials

The data set on which the conclusions of the paper rely are partly included in the manuscript. The study is not finished yet therefore additional data are available on request to corresponding author.

## Additional file

Additional file 1: The questionnaire for the musculoskeletal symptoms. (DOCX 16 kb)

## Abbreviations

2DE: two-dimensional gel electrophoresis
MS: mass spectrometry
MSD: musculoskeletal disorders
VDBP: vitamin D-binding protein
