# Supplementary material for: Biomarkers of systemic inflammation in farmers with musculoskeletal disorders; a plasma proteomic study
Source: BMC Musculoskelet Disord. 2016 May 10;17:206. doi: 10.1186/s12891-016-1059-y (PMC4862124; doi:10.1186/s12891-016-1059-y)
Supplement: Additional file 1: — The questionnaire for the musculoskeletal symptoms. (DOCX 16 kb) [file 12891_2016_1059_MOESM1_ESM.docx]

Movement form signature date running no.

A. QUESTIONS ABOUT DIFFICUTIES IN THE NECK AND/OR SHOULDER/SHOULDER JOINT REGIONS

1. Have you ever (not only occasionally) had difficulty (aches, pains or discomfort) in the neck, shoulder or shoulder joint areas?

no
yes

1. Have you during the past year had difficulty (aches, pains or discomfort) in the neck, shoulder or shoulder joint areas?

no
yes

1. Have you, due to difficulties with the neck, shoulder or shoulder joints at any time been examined or treated by a doctor, physiotherapist or chiropractor?

no
yes

B. QUESTIONS ABOUT DIFFICULTIES IN THE HANDS AND LOWER ARMS

1. Have you ever had difficulties with your hands falling asleep or having a pricking sensa­tion in your hands?

no
yes

1. Have you, due to problems with your hands falling asleep or having a pricking sensation

at any time been examined or treated by a doctor or physiotherapist?

no

yes

1. Have you at any time had difficulties with aches in your hands or arms at night (so that you awoke and had difficulty in sleeping again)?

no

yes

1. Have you, due to problems with nightly pain in your hands and/or arms at any time been examined or treated by a doctor or physiotherapist?

no

yes

1. Have you found that the problems with your hands/arms have (or had) a relation with any particular type of work?

no

yes

Which type of work?

6. Have you been operated or have had any particular treatment for "nerve contraction?" (or other type of nerve problem) in your wrist?

no

yes

C. QUESTIONS ABOUT LOWER BACK PROBLEMS

1. Have you ever had problems with the lower back area (aches, pains or discomfort)? no

yes

1. Have you during the past year had problems with the lower back area (aches, pains or discomfort)?

no

yes

1. Have you, due to problems with the lower back area at any time been examined or treated by a doctor, physiotherapist or chiropractor?

no

yes

1. Have you at any time been sick-listed due to lower back problems? no

yes

1. Have the lower back problems been related to pains radiating down one of your legs (past the knee)?

no

yes

1. Have you, due to problems with ischia’s at any time been examined or treated by a doctor or physiotherapist?

no

yes

D. QUESTIONS ABOUT HIP JOINT PROBLEMS

1. Have you at any time had difficulties due to the hip joints (aches, pain or discomfort)?
   no

yes

1. Have you at any time had difficulties with the groin or the inside of the thigh down to the knee (aches, pains or discomfort)?

no

yes

1. Have you had problems with the hips, groin or thighs (according to questions 1 and/or 2) during the past year?

no
yes

1. Have you at any time been examined or treated by a doctor or physiotherapist for prob­lems with the hips, groin, or thighs (according to question 1 and/or 2)?

no

yes

1. Have you been operated for worn hip joints?
   no

yes

E. QUESTIONS ABOUT KNEE JOINT PROBLEMS

1. Have you ever had difficulties with one or both of your knees (aches, pains or discom­fort)?

no

yes

2. Have you had difficulties with one or both of your knees (aches, pain or discomfort) during the past year?

no

yes

1. Have you, due to difficulties with one or both knees at any time been examined or treated by a doctor or physiotherapist.

no

yes

1. Have you, due to difficulties with one or both knees at any time been sick-listed?
   no

yes

1. Have you been operated for meniscus injury? no

yes

End
